# The dissolution of temporal distance increases risk-taking: experimental evidence

**DOI:** 10.1038/s41598-018-34780-2

**Published:** 2018-11-08

**Authors:** Rafał Muda, Paweł Niszczota, Paweł Augustynowicz, Łukasz Markiewicz

**Affiliations:** 1 Faculty of Economics, Maria Curie-Sklodowska University of Lublin, Lublin, Poland; 2 Department of International Finance, Poznań University of Economics and Business, Poznań, Poland; 3 Department of Experimental Psychology, The John Paul II Catholic University of Lublin, Lublin, Poland; 4 Centre for Economic Psychology and Decision Sciences, Kozminski University, Warsaw, Poland

## Abstract

Earlier research shows that delaying the realization of a lottery (temporal distance) increases risk tolerance. Presumably, this happens because temporal distance protects one from encountering the negative emotions produced when facing risk. However, no study has tested whether people that made a choice in the presence of temporal distance would actually change their decision later on (in the absence of temporal distance), towards the safer choice. To test this, 137 participants were subject to actual temporal distance, consisting of a four-week waiting period. To explore how each participant behaved “in the heat of the moment” (in the absence of temporal distance), we assessed their electrodermal activity and analysed self-description measures of susceptibility to affect. Participants had to choose between 40 lottery pairs (they could win up to the equivalent of about $400 US; the expected payout for each participant was about $12). Results showed that, contrary to expectations, participants tended to choose *riskier* lotteries after the waiting period. The results of an additional experiment suggest that this is not the result of prior exposure to the same set of lotteries, however, interestingly, an exploratory analysis showed that the main effect was driven by the behaviour of male participants. We discuss possible explanations for our surprising main finding and its implications for studies on temporal distance.

## Introduction

Imagine someone who decided to bungee jump. Back home, the person might be confident that he or she will jump, but when on top of the bungee platform, the perception of the situation might change completely, and the person ultimately might refuse to jump. The purpose of this study is to test if people who decide to participate in a risky gamble that will be resolved in the future (e.g., a month after the decision has been made) will stick with their preferred gamble to the moment that the outcome becomes known or if, instead, they would reverse their decision (i.e., “chicken out”^1^). The paradigms used in previous research reflected situations in which subjects made their decision once and could not change it. However, as in the bungee jump case, it is easy to imagine situations in which the initial decision might be changed as time goes by. This warrants a direct investigation of what happens when temporal distance disappears.

Why would someone reverse their decision? On the one hand, the possibility of winning a large amount of money should bring feelings of suspense^2^ and hope^3^ between the decision to participate in the gamble and the moment of risk resolution. If this was the case, most participants will likely stick to their prior decision. On the other hand, the lack of temporal distance^4^ could increase anxiety related with receiving an unfavourable outcome, causing people to reverse their choice. Negativity bias^5^ suggests that, in general, negative affect (e.g., anxiety) will dominate positive affect (e.g., hope), causing people to reverse their decisions.

Our choice of gambles is similar to those made by van Winden *et al*.^6^, who used two kinds of lotteries: (1) with a high probability of success and low payoff and (2) with a low probability of success and high payoff. This distinction is important as these and other researchers revealed that the emotional impact of the gamble depends on the probability of success. Negative anticipatory emotions (e.g., fear or worry) are more important when we deal with a high probability of success, whereas positive emotions (e.g., hope) are more salient in the case of a low probability of success. Additionally, the interplay between positive and negative affect in determining the decision to reverse one’s decisions also gives room for an effect of individual differences in the susceptibility to affect. Thus, we complemented our main analysis by testing how the affective (hope or fear) properties of the lottery influence choices, and if individual differences in susceptibility to positive and negative affect moderate participants’ behaviour.

Our experiment was designed to test what happens when there is actual waiting time (i.e., a four-week delay) between the gamble choice and realization, if people are given the choice to amend their earlier decisions. One of our main goals was to test if the dissolution of temporal distance (i.e., the disappearance of temporal distance between the first and second visit to the laboratory) makes people less risk tolerant. We also sought to verify in a direct way if changes in emotional reactions are connected to the change in risk-taking, and if emotional reactions will be elevated when temporal distance is absent. An important contribution of this research is that we measured the emotional reactions of participants during both their visits at the laboratory, by using self-description and physiological measures. To the best of our knowledge, we are the first to use an objective measure of emotional reactions (i.e., electrodermal activity; EDA) to verify if temporal distance attenuates emotional reactions. The susceptibility to affect was measured via personality traits questionnaires, including the Ten Item Personality Inventory^7^ (TIPI); the Behavioral Inhibition System-Behavioral Approach System scale^8^ (BIS-BAS), and the general Positive and Negative Affect Schedule^9^ (PANAS).

## Results

### Risk-taking propensity

To assess if the presence of temporal distance shifts risk-taking preferences, we analysed our data using a 2 × 2 repeated-measures analysis of variance (ANOVA). The first factor concerned choices made in the initial vs. second visit (round), while the second factor featured “hope” lotteries vs. “fear” lotteries; both factors were within-subject. We scored 1 for each riskier choice and 0 for each safer choice 0 in both lottery conditions. We used the aggregated result from each visit as a risk-taking propensity measure, which showed how often participants chose the riskier lottery from a lottery pair (in a block of 20 lottery pairs, thus the maximum risk-taking score was equal to 20).

**Figure 1:**
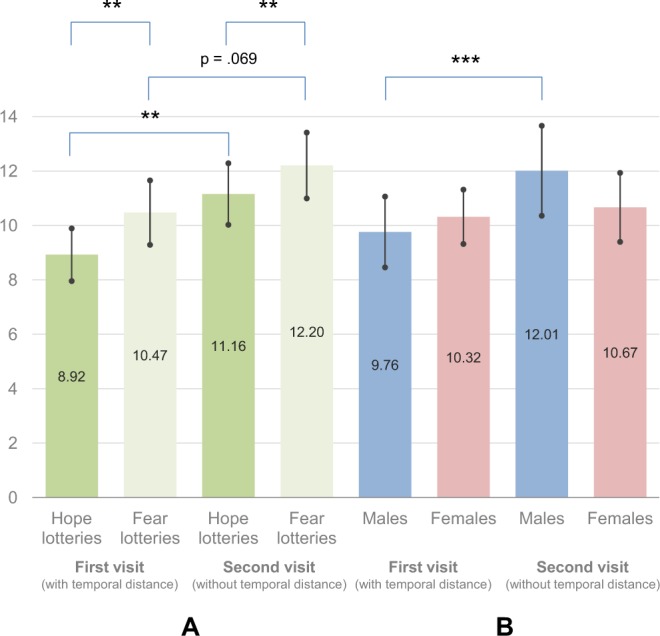
Mean scores of risk-taking during both visits – a comparison of hope and fear lotteries (**A**) and males and females (**B**). The risk-taking score shows the number of risky lotteries chosen (out of 20 pairs of lotteries in each block), and thus a higher score indicates higher risk-taking. Error bars indicate 95% confidence intervals. (***p < 0.001, **p < 0.01).

The results (see Fig. 1A) revealed a significant effect of round condition (F(1, 136) = 5.63, *p* = 0.019 η_p_^2^ = 0.040). Unexpectedly, participants made more risky choices during the second visit (*M* = 11.19, CI 95% [10.18, 12.19]) compared to their initial choices (*M* = 10.17, CI 95% [9.38, 10.95]). This translates into a Cohen’s *d* of 0.408, and should be thus treated as a slightly lower than medium effect size (0.5)^10^. We also observed a main effect concerning the kind of lottery (F(1, 136) = 13.37, *p* < 0.001, η_p_^2^ = 0.091). Subjects chose more risky options in the fear condition (*M* = 11.67, CI 95% [10.69, 12.65]) compared to the hope condition (*M* = 9.69, CI 95% [8.75, 10.63]). There was no round × lottery interaction (F(1, 136) = 1.42, *p* = 0.235, η_p_^2^ = 0.010).

Additionally to the planned analysis, we checked whether sex moderates risk-taking behaviour. To do this, we ran a similar analysis of variance as in the first step – the only difference was sex (male vs. female) added as a between-subject factor. Interestingly, we noticed a significant round × sex interaction (F(1, 133) = 4.63, *p* = 0.033, η_p_^2^ = 0.034). Decomposing the interaction (see Fig. 1B), we observed an increase in risk-taking among men (*M*_*first visit*_ = 9.76, CI 95% [8.46, 11.06] vs. *M*_*second visit*_ = 12.01, CI 95% [10.37, 13.66], F(1, 133) = 10.28, *p* = 0.002, η_p_^2^ = 0.072) but not women (*M*_*first visit*_ = 10.32, CI 95% [9.32, 11.32] vs. *M*_*second visit*_ = 10.67, CI 95% [9.40, 11.93], F(1, 133) = 0.42, *p* = 0.520, η_p_^2^ = 0.003). The difference observed in the male subsample – which corresponds to an effect size (Cohen’s *d*) of 0.557 – was the only statistically significant difference.

### Emotional reactions (state PANAS)

To test the hypothesis that emotional reactivity decreases with temporal distance, we conducted a 2 × 2 repeated-measures ANOVA, with the first factor concerning choices made in the initial vs. second visit, and the second factor concerning affect, with state PANAS items with positive valence vs. state PANAS items with negative valence. Both factors were within-subject.

Results show a significant effect of the round condition (F(1, 131) = 9.19, *p* = 0.003 η_p_^2^ = 0.066), as expected. The level of emotional reactions was higher during the second visit (*M* = 2.68, CI 95% [2.60, 2.76]) than during the first visit (*M* = 2.56, CI 95% [2.51, 2.62]).

Importantly, we also observed a significant time × affect interaction (F(1, 131) = 7.57, *p* = 0.007 η_p_^2^ = 0.055). Decomposing the interaction, we noticed that an increase in emotional reactions was present only in the case of negative feelings (*M*_*first visit*_ = 1.49, CI 95% [1.40, 1.58] vs. *M*_*second visit*_ = 1.70, CI 95% [1.58, 1.83], F(1, 131) = 17.06, *p* < 0.001, η_p_^2^ = 0.115).

**Figure 2:**
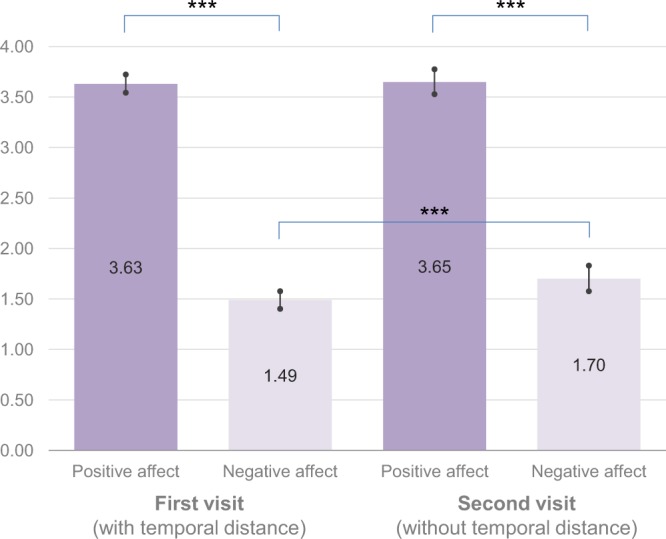
Mean scores of emotional reactions measured with PANAS for both positive and negative affect. Error bars indicate 95% confidence intervals. (***p < 0.001).

There was also a main effect of affect (F(1, 131) = 685.05, *p* < 0.001 η_p_^2^ = 0.839); participants indicated higher ratings of positive affect (*M* = 3.64, CI 95% [3.55, 3.74]) than of negative affect (*M* = 1.60, CI 95% [1.50, 1.69]). See Fig. 2 for a summary of the results.

As in the case of risk-taking propensity, we conducted an additional analysis including sex as a between-subject factor. In the case of PANAS, however, we did not observe any statistically significant gender differences.

### Emotional reactions (physiology)

To assess the changes in the physiological reactions, we conducted a 2 × 2 repeated-measures ANOVA, in which the first factor concerned choices made in the initial vs. second visit, while the second one concerned the type of lottery, i.e. hope lotteries vs. fear lotteries. Both factors were within-subject.

As in the case of the PANAS ANOVA, we observed a main effect of the round condition (F(1, 118) = 8.06, *p* = 0.005, η_p_^2^ = 0.064). The level of physiological reactions was higher during the second visit (*M* = 0.26, CI 95% [0.22, 0.29]) compared to the first one (*M* = 0.21, CI 95% [0.19, 0.24]).

**Figure 3:**
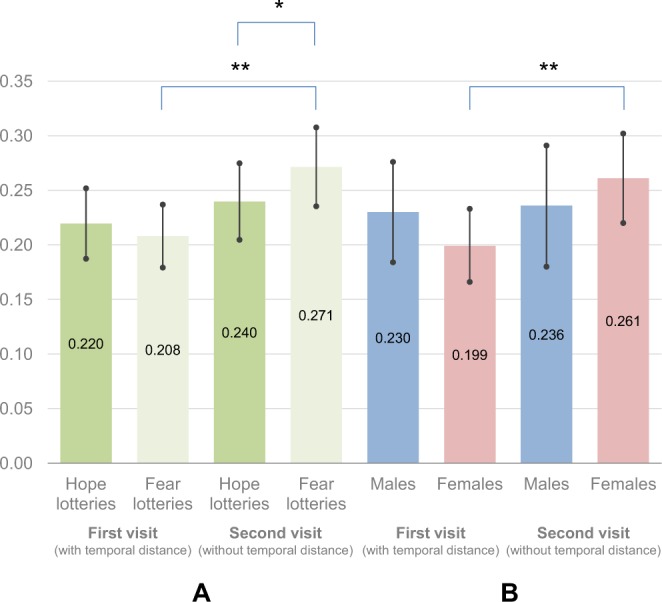
Mean skin conductance responses during both visits – a comparison of hope and fear lotteries (**A**) and males and females (**B**). Error bars indicate 95% confidence intervals. (**p < 0.01, *p < 0.05).

Results also revealed a significant time × lottery kind interaction (F(1, 118) = 7.59, *p* = 0.007 η_p_^2^ = 0.060). Decomposing the interaction, we observed significant differences between visits only for fear lotteries (*M*_*first visit*_ = 0.21, CI 95% [0.18, 0.24] vs. *M*_*second visit*_ = 0.27, CI 95% [0.24, 0.31], F(1, 118) = 12.90, *p* < 0.001, η_p_^2^ = 0.099). The participants’ physiological reactions are reported in Fig. 3A, which shows the mean skin conductance responses of participants during both visits, separately for hope lotteries and fear lotteries.

In the case of physiological reactions we also conducted an additional analysis of variance including sex as a between-subject factor. We observed a marginally significant round × sex interaction (F(1, 115) = 3.20, *p* = 0.076, η_p_^2^ = 0.027). Decomposing the interaction (see Fig. 3B), we observed an increase in physiological reactions among women (*M*_*first visit*_ = 0.199, CI 95% [0.166, 0.233] vs. *M*_*second visit*_ = 0.261, CI 95% [0.220, 0.302], F(1, 115) = 11.23, *p* = 0.001, η_p_^2^ = 0.089) but not men (*M*_*first visit*_ = 0.230, CI 95% [0.184, 0.276] vs. *M*_*second visit*_ = 0.236, CI 95% [0.180, 0.291], F(1, 133) = 0.06, *p* = 0.810, η_p_^2^ < 0.001). This was the only statistically significant difference.

### Change in risk-taking and individual differences in susceptibility to affect

We used regressions (see Table 1) to assess if susceptibility to positive and negative affect (operationalized via various measures) had an influence on the change in behaviour between the first and second visit. The dependent variable was defined as the difference between switches from risky to safe lotteries and switches from safe to risky lotteries, and thus measured the net tendency to reduce risk. Generally speaking, we did not find evidence that susceptibility to either positive affect (i.e., extraversion, BAS, or trait PANAS items with a positive valence) or negative affect (i.e., neuroticism, BIS, or trait PANAS items with a negative valence) had an impact on the change in behaviour (choice of lotteries) between both visits.

**Table 1:** Regressions showing the relationship between change in risk-taking and individual differences in susceptibility to affect.

	All lotteries	Hope lotteries	Fear lotteries
Neuroticism	−0.009 (0.020)	−0.003 (0.022)	−0.015 (0.026)
Negative affect (PANAS)	−0.026 (0.038)	−0.114** (0.040)	0.063 (0.049)
Behavioral Inhibition System	−0.024 (0.057)	0.028 (0.061)	−0.076 (0.074)
Extraversion	0.014 (0.019)	−0.006 (0.020)	0.033 (0.024)
Positive affect (PANAS)	−0.042 (0.057)	−0.042 (0.062)	−0.042 (0.074)
Behavioral Approach System	0.031 (0.063)	0.122 (0.068)	−0.061 (0.082)
Female participant	0.101* (0.047)	0.063 (0.050)	0.139* (0.060)
General risk tolerance	−0.011 (0.013)	−0.007 (0.013)	−0.014 (0.016)
Risk tolerance (gambling)	−0.040 (0.028)	−0.044 (0.03)	−0.037 (0.036)
*N*	135	135	135
*Adjusted R* ^*2*^	0.016	0.052	0.025
*F-statistic*	1.24	1.82	1.38

Standard errors are shown in parentheses. (**p < 0.01, *p < 0.05).

### Correlation between risk-taking and state PANAS

We complemented our analyses with two exploratory analyses, that were meant to assess whether the number of risky lotteries that were chosen in each block was correlated with either state PANAS (i.e., a self-description measure) or skin conductance responses (i.e., a physiological measure). Results of a pairwise correlation analysis between the state PANAS data (for both subscales) and risk-taking propensity (for both blocks) – see Table 2 – indicated that during the first visit, a higher level of positive affect was connected with a higher level of risk-taking in the case of hope lotteries (*r*(132) = 0.36, *p* < 0.001), whereas a higher level of negative emotions was connected with a lower level of risk-taking in the case of fear lotteries (*r*(132) = −0.20. *p* = 0.025).

**Table 2:** Pairwise correlations between PANAS results (for both the positive and negative subscale) and risk-taking propensity during the first and second visit.

	PANAS positive	PANAS negative	Risk-taking hope	Risk-taking fear
**First visit**				
PANAS positive	1			
PANAS negative	−**0.261** **(0.003)**	1		
Risk-taking hope	**0.361** **(<0.001)**	−0.077 (0.382)	1	
Risk-taking fear	0.117 (0.183)	−**0.195** **(0.025)**	**0.229** **(0.007)**	1
**Second visit**				
PANAS positive	1			
PANAS negative	−**0.209** **(0.016)**	1		
Risk-taking hope	**0.226** **(0.009)**	−**0.218** **(0.012)**	1	
Risk-taking fear	**0.200** **(0.021)**	−0.102 (0.246)	**0.521** **(<0.001)**	1

*p*-values are shown in parentheses.

Interestingly, during the second visit, the relationship between the level of emotional reactions and risk-taking changed. The propensity for risk-taking in the case of hope lotteries was positively associated with positive affect (*r*(132) = 0.23. *p* = 0.009) and negatively connected with negative affect (*r*(132) = −0.22, *p* = 0.012), whereas risk-taking propensity in the case of fear lotteries was positively related with positive affect (*r*(132) = 0.20, *p* = 0.021).

### Correlation between risk-taking and physiology

Results of a pairwise correlation between EDA and risk-taking (in each block) revealed that physiological reactions were not connected with the propensity for risk-taking (during either visit; *p*s > 0.131).

### The role of prior exposure to the lotteries

A reasonable argument that can be made is that our research design has a possible confound, due to the fact that the choices made during the second visit at the laboratory are not only made with the expectation that they will be resolved immediately (and not after a four-week waiting period), but are also made one after the other. In other words, participants that made the decisions during the second visit were exposed to the same set of lotteries for the second time, which in itself could change their behaviour. To overcome this limitation of the design, we performed another (“control”) experiment, in which participants had to choose between the same set of lotteries as we used earlier, but contrary to the earlier design, they were told that the lotteries will be resolved immediately. After making their choices, participants completed a filler task, after which they were (unexpectedly) told to choose from the same set of lotteries the second time (and were told that for each block we will randomly choose one lottery from all the lotteries they chose during both rounds). This allowed us to test the effect of a prior exposure to the same set of lotteries. Eighty-five participants were recruited, which allowed us to obtain 95% power to detect an effect size of f = 0.20 (α = 0.05, number of groups = 2, number of measurements = 2, correlation among repeated measures = 0.5, nonsphericity correction = 1). Results showed only one significant difference – a main effect of block (F(1, 84) = 23.43, *p* < 0.001, η_p_^2^ = 0.218): participants took more risk in the fear block (*M* = 12.41, CI 95% [10.90, 13.91]) than in the hope block (*M* = 8.97, CI 95% [7.35, 10.59]). This is in line with the result observed in the original experiment, which suggests that our findings are robust. Crucially, in contrast to the original experiment, we did not observe a significant increase in risk-taking during the second exposure to the lotteries (*M*_*first exposure*_ = 10.58, CI 95% [9.19, 11.97] vs. *M*_*second exposure*_ = 10.80, CI 95% [9.33, 12.28], F(1, 84) = 0.38, *p* = 0.541, η_p_^2^ = 0.004). The block × exposure interaction was also not significant (F(1, 84) = 2.51, *p* = 0.117, η_p_^2^ = 0.029). We interpret this as inconsistent with the interpretation that exposure to the same set of lotteries (that happened during the second visit of our earlier design) is responsible for the increase in risk-taking that we observed in the second visit of our original experiment.

## Discussion

The current work focused on testing how people behave when given the opportunity to reverse the initial decisions that they made in the presence of actual temporal distance. Based on previous findings^11–13^, we conducted this study with the expectation that the change in proximity to the realization of the lottery (between the first and second round of choices, i.e. made in the presence and absence of temporal distance, respectively) will cause a change (reduction) in risk-taking. However, the data did not support the hypothesis that participants will shift to less risky gambles when given the chance, but instead revealed that participants took on more risk at the moment of risk resolution compared to choices made in the presence of temporal distance. This seems to suggest that the absence of temporal distance does not necessarily lead to less risk-taking behaviour, and extends our knowledge about the connection between temporal distance and the propensity to take risks. Our findings should not be interpreted as contradictory to what was shown in previous research that used a between-subjects design contrary to our within-subjects design, given the differences in approaches. Rather, our approach shows that the effect of temporal distance on risk-taking is complex and intriguing, with many potential underlying mechanisms (cognitive, emotional, or both).

We offer two possible explanations for why participants actually increased risk-taking, contrary to expectations. The first relates to mood maintenance. Individuals in a negative mood seek riskier options, because obtaining a high reward might improve their mood^14,15^. This would be in line with our observation showing that during the second visit (i.e., without temporal distance), the level of negative emotional reactions increased, whereas positive emotional reactions did not change. Note, however, that we did not observe any relationship between change in risk-taking and individual differences in susceptibility to affect. That is, participants that were susceptible to negative affect (i.e., those who should more easily enter negative moods) did not differ in their risk-taking behaviour from other participants. Thus, our data is not entirely consistent with this explanation, and makes the next explanation more plausible.

The second possible explanation relates to the affective deconstruction of the weighting function^16^, which implies that large jumps at the ends of the function depend on the affective reactions elicited by possible outcomes. More precisely, affect-rich prizes should elicit greater feelings of hope and fear than affect-poor prizes and, therefore, larger jumps should occur at the ends of the probability weighting function (intermediate probabilities, however, should be less sensitive to change). Considering the increase in emotional and physiological reactions during the second visit, it is possible that participants perceived outcomes as more affective after the waiting period. If this was true, based on Rottenstreich and Hsee^16^, this should result in an increase in the probability weighting for hope lotteries (with a small chance of winning) and a decrease in the probability weighting for fear lotteries (with a small chance of losing). In other words, participants should perceive that winning 1,500 PLN with a 1% chance as more likely during the second visit than during the first one; thus, they should be more willing to make such a choice. On the other hand, winning 15 PLN with a 99% chance should be perceived as less likely during the second visit, and thus participants should be more likely to make the riskier choice.

Another contribution of our research is that by using self-reported and physiological measures, we experimentally confirmed previous findings, namely that the timing of the resolution attenuates the level of emotional reactions^6,13^. We also confirmed van Winden *et al*.’s^6^ notion that positive emotional reactions are connected with higher risk-taking for lotteries that induce feelings of hope, whereas negative emotional reactions are connected with lower risk-taking for lotteries that induce feelings of fear. This only happened, however, in the presence of temporal distance; without it, we observed that positive emotional reactions were related with higher risk-taking for both types of lotteries, and negative emotional reactions were associated only with lower risk-taking for hope lotteries.

These results might be explained by the different emotional states experienced or anticipated while making a decision in the presence or absence of temporal distance. Usually, we are more willing to delay the resolution of an event that gives us a small probability of obtaining a large gain^3^, as this enables us to feel hopeful (an extreme example of this is buying a lottery ticket). When temporal distance disappears, the situation changes. Risk is to be resolved immediately, leaving no room for additional utility derived from experiencing positive feelings while waiting. Additionally, the post-outcome anticipated emotions (e.g., regret) become more salient when time goes by^13^, and we focus both on positive and negative consequences of our decision^17^.

Our results seem to be in line with the cold/hot empathy gap^18^, which states that in “cold” emotional states, we rather poorly predict how we will behave or what we will prefer in a “hot” state. Based on our study, we can conclude that present feelings and experiences are poor predictors of future behaviour, and if we want to know exactly how someone will behave in a specific situation that is driven by a concrete mixture of emotions, we should test this behaviour directly. Thus, the presence of actual temporal distance is needed if we want to study risk preferences about future prospects (and future preferences in general). This idea is in line with the prospection mechanism^19^, which assumes that our predictions of future reactions to an event are influenced by both the anticipation of the future event and present contextual factors (e.g., other events occurring in the present moment, present thoughts, bodily experiences).

Importantly, as Gilbert and Wilson noticed, on the basis of our present experiences, we can reliably predict future experiences only if *“(i) our simulation of the event at T*_1_ *exerts the same influence on our hedonic experience at T*_1_ *as our perception of the event at T*_2_ *exerts on our hedonic experience at T*_2_*, and (ii) contextual factors at T*_1_ *exert the same influence on our hedonic experience at T*_1_ *as contextual factors at T*_2_ *exert on our hedonic experience at T*_2_ (p. 1352)”^19^. Thus, people who went to Las Vegas and decided to play only for the lowest stakes might change their attitude after entering the casino. Similarly, people who felt sick and made a doctor’s appointment might later change their mind and go directly to work (risking a further deterioration in health).

It is worth discussing what we learn from the exploratory analyses that were meant to assess possible gender effects and differences in electrodermal activity in lotteries that either cue hope or fear. An analysis of gender differences revealed that the increase in risk-taking appeared to be driven by male participants. The main takeaway point from this finding is that future studies on the effect of temporal distance on risk-taking should be cognizant of possible gender effects. The literature would benefit from attempts to find the causes of these differences (if they re-emerge), as our research presents only a partial explanation for this unexpected finding. A closer inspection of the data shows that after the dissolution of temporal distance SCRs increased only in fear lotteries and only in female participants, and thus one can speculate that male participants were better at fear inhibition in the absence of temporal distance, consistent with studies that show sex differences in fear inhibition^20^. However, we do not have direct evidence that this was the reason for the increase in risk-taking. In light of the lack of evidence that male participants were more emotionally engaged during the second visit, it is plausible that male participants might have adopted some “rational” strategy that influenced risk-taking (which could have been facilitated by fear inhibition). For example, during the second visit male participants could have perceived that the costs connected with obtaining the reward have increased, and might have compensated this by choosing riskier lotteries, that had a high expected reward. We have conducted an additional experiment that showed that the increase in risk-taking is not merely due to prior exposure to the same set of lotteries, but leave it to other researchers to determine the exact mechanisms that might lead to risk-taking being elevated and not attenuated in the absence of temporal distance.

We are aware that our findings might not generalize to all kinds of situations with delayed risk resolution. In our study, we only used lotteries with possible gains, and it is possible that the possibility of losses will make people behave in a risk-averse way. In line with this is the idea that people differently perceive the attractiveness of delayed gambles that are positively or negatively skewed^21^. It would be beneficial to explore how the possibility of a loss and the opportunity to change initial decisions will make people behave.

## Methods

### Participants

One hundred and thirty-seven people took part in all stages of the experiment, and only these data were analysed (*M*_*Age*_ = 23.98, *SD* = 5.34, 63% women). Participants were recruited through the Flow Research Center subject pool and by advertising on Maria Curie-Sklodowska University web pages. Although the study was advertised as an investigation into the factors affecting financial decision-making in which participants could win money depending on their choices, initially participants did not know the real purpose of the experiment. We told all participants that the study consisted of an online survey and two visits in the laboratory, and that besides the possibility of winning money based on their choices, each would be paid for showing up for the second laboratory visit (10 PLN, or about $2.70).

This study was approved by the Kozminski University Ethics Committee and was carried out in accordance with relevant guidelines. Before data collection, all participants were informed about the study protocol and gave written consent to take part in the study.

### Procedure

During the first stage of the study, participants completed an online survey meant primarily to assess their personality traits and risk tolerance level. The survey featured brief demographic questions, followed by the TIPI, Polish adaptation^22^; BIS-BAS, Polish adaptation^23^; general PANAS^9^; general risk-taking scale^24^; and DOSPERT gambling subscale^25^. Participants then registered for the laboratory phase.

The second stage comprised two laboratory visits. At the beginning of the study, we informed participants that they would choose between pairs of lotteries that would be resolved in four weeks. We also told them we would measure the EDA during the study (see the EDA measurement section for more details). Afterward, they provided their consent for taking part in the experiment, and we began measuring their risk-taking propensity.

In this task, subjects made 40 choices between pairs of lotteries grouped in two blocks: 20 pairs were supposed to induce feelings of hope and 20 feelings of fear (for more details, see the risk-taking lottery task section). Participants were told that during the second visit, we would draw one of their choices from each block and resolve it; if they won, they would be paid. We wrote the instructions with the intention of making the participants feel that their decisions are important, as they were connected with actual payoffs (i.e., that they were not merely hypothetical choices). More specifically, we used the following instructions: *“In a moment, we will present to you pairs of lotteries (one pair on each screen). (…) Altogether, we will present 40 lottery pairs, in two blocks containing* 2*0 pairs each. The lotteries will be played out 4 weeks from now. (…) From each block we will randomly select one lottery, the result of which will determine your payoff.”* After completing their choices, we asked subjects to indicate their actual feelings on the PANAS scales.

During the second visit, subjects completed the same task as in the initial visit. At the beginning, we told the participants that they made choices four weeks prior but could make choices once again. Because we did not want to prime participants in any way, we kept our instructions superficial and did not remind the subjects of their initial choices. After the instructions, we began the study task. At the end, we drew the lotteries and paid the winnings.

### Measures

#### Risk-taking lottery task

The lottery task comprised two blocks of lottery pairs. The aim of the lottery blocks was to induce specific emotional reactions associated with a decision, namely feelings of hope and fear. We constructed lotteries similarly to Ahlbrecht and Weber^26^ and based on Rottenstreich and Hsee’s^13^ notion that whenever the probability of winning is greater than 0, we deal with situations in which some hope exists (compared to situations when the probability of winning is equal to 0). When the probability of winning is less than 1, we deal with situations when some fear exists (compared to situations when the probability of winning is equal to 1). Moreover, any changes in the case of intermediate probabilities induce a relatively small emotional impact. This is also in line with Ahlbrecht and Weber’s^26^ and Wu’s^27^ notions about anticipatory emotions, which assume prospects with low probability and high payoff should elicit positive emotions (e.g., hope), whereas prospects with high probability and low payoff should elicit negative feelings (e.g., fear).

Taking this into account, we created 20 lottery pairs in each block with equal expected value of alternatives. In the block inducing feelings of hope in each pair, one lottery was an option with a high potential for winning but low probability of doing so (e.g., 1% for 1,500 PLN), and the second lottery was an option with an intermediate payoff and probability of winning (e.g., 50% for 30 PLN). The aim of this block was to induce feelings of hope connected with the high possibility of winning. Likewise, in the block inducing feelings of fear, in each pair, one lottery was an option with a low potential for winning and a very high probability of doing so (e.g., 99% for 15 PLN), and the second lottery was an option with an intermediate payoff and probability of winning (e.g., 50% for 30 PLN). The aim of this block was to induce feelings of fear connected with the possibility of not winning. To see the full list of lottery pairs, see Supplementary Table S1.

#### EDA measurement and processing procedure

A constant current EDA measurement system (BIOPAC MP36, Biopac System Inc., Goleta, USA) was used to acquire skin conductance response (SCR) data. The data were sampled at 2000 Hz with flat Ag-AgCl electrodes attached to the intermediate phalanges of the index and middle fingers of the left, non-dominant hand. Electrodes were connected at least 10 minutes prior to the start of the experiment to allow EDA levels to stabilize^28^.

EDA data were analysed using custom Matlab^®^ R2014b scripts in conjunction with Ledalab (V3.4.9) toolbox^29^. Data processing included smoothing continuous data using an adaptive smoothing algorithm. Then data were visually inspected for moving and other artefacts. Artefactual data components of no more than 1 second time windows were corrected by means of interpolation. Artefact-free data series were subject to continuous decomposition analysis (CDA) to allow for extraction of phasic and tonic SC data.

A set of six initial starting points were used to calculate a best fit of Tau1 and Tau2 parameters of bi-exponential Bateman function. After CDA model fitting, datasets were inspected for compound error measure. Compound error that exceeded a 3*SD threshold or high negativity error ratio resulted in rejecting datasets from the analysis.

The analysis included 119 of 137 complete and artefact-free datasets. A minimum amplitude threshold criterion of 0.01 μS was used for SCR measures. Afterward, standardized SCR measures were computed for fear and hope blocks separately and divided by the corresponding baseline. For baseline SCR activity, a period of 120 seconds prior to the start of the experiment was used.

## Electronic supplementary material

Supplementary Table S1

## Data Availability

The datasets generated during and/or analysed during the current investigation are available from the corresponding author on reasonable request.
