# Supplementary material for: The dissolution of temporal distance increases risk-taking: experimental evidence
Source: Sci Rep. 2018 Nov 8;8:16565. doi: 10.1038/s41598-018-34780-2 (PMC6224535; doi:10.1038/s41598-018-34780-2)
Supplement: Supplementary file 1 — Supplementary Table S1 [file 41598_2018_34780_MOESM1_ESM.docx]

**Supplementary materials**

**The dissolution of temporal distance increases risk-taking: experimental evidence**

Rafał Muda, Paweł Niszczota, Paweł Augustynowicz, Łukasz Markiewicz

**Supplementary Table S1. Pairs of lotteries used in this study**

| **Lottery pair** | **Lottery A** | | | | **Lottery B** | | | |
| --- | --- | --- | --- | --- | --- | --- | --- | --- |
|  | **Win** | | **Loss** | | **Win** | | **Loss** | |
|  | **Prob.** | **Outcome (PLN)** | **Prob.** | **Outcome (PLN)** | **Prob.** | **Outcome (PLN)** | **Prob.** | **Outcome (PLN)** |
| **Hope lotteries (N=20)** | | | | | | | | |
| 1 | 1% | 1500 | 99% | 0 | 30% | 50 | 70% | 0 |
| 2 | 2% | 750 | 98% | 0 | 35% | 43 | 65% | 0 |
| 3 | 3% | 500 | 97% | 0 | 40% | 38 | 60% | 0 |
| 4 | 4% | 375 | 96% | 0 | 45% | 33 | 55% | 0 |
| 5 | 5% | 300 | 95% | 0 | 50% | 30 | 50% | 0 |
| 6 | 1% | 1500 | 99% | 0 | 55% | 27 | 45% | 0 |
| 7 | 2% | 750 | 98% | 0 | 60% | 25 | 40% | 0 |
| 8 | 3% | 500 | 97% | 0 | 65% | 23 | 35% | 0 |
| 9 | 4% | 375 | 96% | 0 | 70% | 21 | 30% | 0 |
| 10 | 5% | 300 | 95% | 0 | 75% | 20 | 25% | 0 |
| 11 | 1% | 1500 | 99% | 0 | 80% | 19 | 20% | 0 |
| 12 | 2% | 750 | 98% | 0 | 30% | 50 | 70% | 0 |
| 13 | 3% | 500 | 97% | 0 | 35% | 43 | 65% | 0 |
| 14 | 4% | 375 | 96% | 0 | 40% | 38 | 60% | 0 |
| 15 | 5% | 300 | 95% | 0 | 45% | 33 | 55% | 0 |
| 16 | 1% | 1500 | 99% | 0 | 50% | 30 | 50% | 0 |
| 17 | 2% | 750 | 98% | 0 | 55% | 27 | 45% | 0 |
| 18 | 3% | 500 | 97% | 0 | 60% | 25 | 40% | 0 |
| 19 | 4% | 375 | 96% | 0 | 65% | 23 | 35% | 0 |
| 20 | 5% | 300 | 95% | 0 | 70% | 21 | 30% | 0 |
| **Fear lotteries (N=20)** | | | | | | | | |
| 1 | 99% | 15 | 1% | 0 | 15% | 100 | 85% | 0 |
| 2 | 98% | 15 | 2% | 0 | 20% | 75 | 80% | 0 |
| 3 | 97% | 15 | 3% | 0 | 25% | 60 | 75% | 0 |
| 4 | 96% | 16 | 4% | 0 | 30% | 50 | 70% | 0 |
| 5 | 95% | 16 | 5% | 0 | 35% | 43 | 65% | 0 |
| 6 | 99% | 15 | 1% | 0 | 40% | 38 | 60% | 0 |
| 7 | 98% | 15 | 2% | 0 | 45% | 33 | 55% | 0 |
| 8 | 97% | 15 | 3% | 0 | 50% | 30 | 50% | 0 |
| 9 | 96% | 16 | 4% | 0 | 15% | 100 | 85% | 0 |
| 10 | 95% | 16 | 5% | 0 | 20% | 75 | 80% | 0 |
| 11 | 99% | 15 | 1% | 0 | 25% | 60 | 75% | 0 |
| 12 | 98% | 15 | 2% | 0 | 30% | 50 | 70% | 0 |
| 13 | 97% | 15 | 3% | 0 | 35% | 43 | 65% | 0 |
| 14 | 96% | 16 | 4% | 0 | 40% | 38 | 60% | 0 |
| 15 | 95% | 16 | 5% | 0 | 45% | 33 | 55% | 0 |
| 16 | 99% | 15 | 1% | 0 | 50% | 30 | 50% | 0 |
| 17 | 98% | 15 | 2% | 0 | 15% | 100 | 85% | 0 |
| 18 | 97% | 15 | 3% | 0 | 20% | 75 | 80% | 0 |
| 19 | 96% | 16 | 4% | 0 | 25% | 60 | 75% | 0 |
| 20 | 95% | 16 | 5% | 0 | 30% | 50 | 70% | 0 |
